# Endoscopic transpapillary gallbladder stenting vs percutaneous cholecystostomy for managing acute cholecystitis: Nationwide propensity score study

**DOI:** 10.1055/a-2521-0084

**Published:** 2025-02-26

**Authors:** Chun-wei Pan, Daryl Ramai, Azizullah Beran, Yichen Wang, Yuting Huang, John Morris

**Affiliations:** 1 25430 Internal Medicine, Cook County Hospital, Chicago, United States; 2 1861 Gastroenterology, Hepatology, and Endoscopy, Brigham and Women's Hospital, Boston, United States; 3 1772 Gastroenterology and Hepatology, Indiana University, Bloomington, United States; 4 14640 Internal Medicine, University of Pennsylvania Perelman School of Medicine, Philadelphia, United States; 5 156400 Gastroenterology & Hepatology, Mayo Clinic in Florida, Jacksonville, United States; 6 14434 Gastroenterology and Hepatology, University of Utah Health, Salt Lake City, United States

**Keywords:** Pancreatobiliary (ERCP/PTCD), ERC topics, Endoscopic ultrasonography, Biliary tract

## Abstract

**Background and study aims:**

Cholecystectomy is the standard treatment for acute cholecystitis, but it may not be suitable for all patients. For those who cannot undergo surgery, a percutaneous cholecystostomy tube (PCT) and ERCP-guided transpapillary gallbladder drainage are viable options. We aimed to perform a nationwide study to assess 30-day readmission rates, adverse events (AEs), and mortality rates in these two cohorts.

**Patients and methods:**

We conducted a nationwide cohort study using data from the Nationwide Readmissions Database (NRD) from 2016 to 2019. We identified patients with acute cholecystitis during the index admission who underwent either PCT or ERCP-guided gallbladder drainage. Propensity score matching along with multivariable regression was used to compare cohorts.

**Results:**

During the study period, 3,592 patients (average age 63.0 years) underwent endoscopic drainage, whereas 80,372 patients (average 70.8 years) underwent Interventional Radiology drainage. Utilizing multivariate Cox regression analysis, compared with ERCP, PCT had a higher risk for 30-day readmission (adjusted hazard ratio [aHR] 1.47; 95% confidence interval [CI] 1.27 to 1.71; *P* < 0.001). The PCT group had a significantly higher rate of readmission for acute cholecystitis compared with the ERCP group (2.72% vs 0.86%; *P* < 0.005). Cox proportional hazard ratio showed a 3.41-fold increased risk (95% CI 1.99 to 5.84) for readmission in the PCT group. ERCP was consistently associated with lower rates of post-procedural AEs compared with PCT including acute hypoxemic respiratory failure ( *P* < 0.001), acute renal failure ( *P* < 0.001), shock ( *P* < 0.001), and need for blood transfusions ( *P* < 0.001).

**Conclusions:**

Our nationwide analysis revealed that ERCP-guided gallbladder drainage should be the preferred approach for managing acute cholecystitis when unfit for surgery.

## Introduction

Acute cholecystitis is a common condition that typically requires surgical intervention. However, in high-risk patients who are poor surgical candidates, alternative drainage methods are necessary 1 . Two primary techniques for non-surgical gallbladder drainage include endoscopic retrograde cholangiopancreatography (ERCP)-guided transpapillary gallbladder drainage and percutaneous cholecystostomy (PTC) 2 .

PTC has long been the standard approach for non-surgical management of acute cholecystitis, offering high technical success rates and immediate symptom relief 3 . However, it requires external drainage, which can lead to patient discomfort and complications such as drain dislodgement or infection. ERCP-guided drainage is a minimally invasive alternative, allowing for internal drainage without need for external catheters 4 5 . Although endoscopic ultrasound-guided drainage (EUS-GB) has grown in popularity in the last few years, it is limited to tertiary clinical centers and expert endoscopists. Thus, ERCP-guided drainage is an appealing clinical option in areas with limited resources.

Although both techniques have demonstrated efficacy in managing acute cholecystitis, comparative outcomes between ERCP-guided drainage and PTC remain unclear, particularly in terms of population-level readmission rates, mortality, and procedure complications. Previous studies have been limited by small sample sizes or single-center designs, potentially limiting their generalizability 2 6 7 .

Despite use of ERCP-guided gallbladder drainage and PTC for acute cholecystitis in high-risk patients, large-scale comparative data on their outcomes remain limited. This study aimed to evaluate 30-day readmission rates, in-hospital mortality, and complications associated with these two techniques using the Nationwide Readmissions Database (NRD). By employing propensity score matching, we seek to provide a comprehensive, nationally representative assessment of these gallbladder drainage methods to inform clinical decision-making.

## Patients and methods

### Study design and data source

We conducted a retrospective cohort study using data from the NRD for the years 2016 to 2019. The NRD, developed and maintained by the Healthcare Cost and Utilization Project (HCUP) from the Agency for Healthcare Research and Quality, is the largest publicly available, nationally representative all-payer database for inpatient care in the United States. In 2019, the NRD included approximately 18 million discharges from 2,430 hospitals across 28 states, representing 60% of the total US resident population and 57% of all hospitalizations in the country.

The NRD collects data on patient demographics, diagnoses, procedures, and resource utilization from all HCUP hospital partners. Hospitals are stratified by ownership/control, bed size (small, medium, and large), teaching status, urban/rural location, and patient location according to National Center for Health Statistics (NCHS) Urban-Rural destination. Each discharge is weighted to ensure that the NRD is nationally representative. The NRD is a unique resource for studying hospital readmissions because it allows researchers to track patients across different hospitalizations within the same state and year. To allow for unbiased analysis of unplanned all-cause 30-day readmissions, we excluded patients who died during the index admission and whose discharges occurred in December because they would not have a full 30-day follow-up period.

### Study population and variables

We identified patients with acute cholecystitis during index admission who underwent either ERCP-guided gallbladder drainage or PTC using ICD-10-CM and ICD-10-PCS codes (K81.0, K80.63, K80.62, K80.43, K80.42, K80.00, K80.01 for acute cholecystitis; 0F9480Z, 0F948ZX, 0F948ZZ, 0F954ZX, 0F957ZX, 0F958ZX, 0F964ZX, 0F967ZX, 0F968ZX, 0F977ZX, 0F978ZX, 0F988ZX, 0F997ZX, 0F998ZX, 0F9C80Z, 0F9980Z, 0FC58ZZ, 0FC68ZZ, 0FC78ZZ, 0FR58JZ, 0FR68JZ, 0FR78JZ, 0FR88JZ, 0FR98JZ for ERCP-guided gallbladder drainage; 0F9430Z, 0F943ZX, 0F943ZZ, 0F753DZ, 0F753ZZ, 0F763DZ, 0F763ZZ, 0F773DZ, 0F773ZZ, 0F783DZ, 0F783ZZ, 0F793DZ, 0F793ZZ, 0F993ZZ, 0F994ZZ, 0FC53ZZ, 0FC54ZZ, 0FC63ZZ, 0FC64ZZ, 0FC73ZZ, 0FC74ZZ, 0FC83ZZ, 0FC84ZZ, 0FC93ZZ for PTC). Patient-level data included age, sex, income in patient zip code, procedures, discharge disposition, length of stay, and total hospitalization charges. Hospital-level data included teaching status, bed size, and urban/rural location. The Elixhauser comorbidity score was calculated for each patient.

### Outcomes

The primary outcome was the 30-day all-cause readmission rate, defined as a second admission to the same or another hospital within 30 days of index admission discharge. If multiple readmissions occurred within 30 days, only the first was captured. Secondary outcomes included procedures performed during the index admission, in-hospital mortality rates of index admission, length of stay, total hospitalization charges, and independent predictors of 30-day readmission. Additional secondary outcomes included rates of post-procedure adverse events (AEs) such as acute hypoxemic respiratory failure, acute renal failure, shock, need for blood transfusions, need for mechanical intubation, and lower gastrointestinal bleeding. In addition, we evaluated specific biliary-related events such as acute cholecystitis, biliary pancreatitis, choledocholithiasis, and acute cholangitis.

In addition, we performed a sensitivity analysis using propensity score matching to ensure comparability between treatment groups. We employed the nearest-neighbor matching method, matching patients on key variables including age, gender, hospital characteristics (such as teaching status and bed size), and Charlson Comorbidity Index. This approach allowed us to balance covariates between groups and reduce potential confounding, ensuring that compared groups had similar baseline characteristics and a more robust estimation of treatment effects. Covariates were visualized using the two-way plot ( **Supplementary Fig. 1** ).

### Statistical analysis

Descriptive statistics were used to summarize patient and hospital characteristics. Categorical variables were compared using Chi-square or Fisher's exact test, whereas continuous variables were compared using Student's *t* -test. Multivariable Cox regression analysis was performed to identify independent predictors of 30-day readmission, controlling for potential confounders. Multivariable regression models were used to adjust for confounders and were built using the following method. Univariable regression analyses on possible confounding factors were used to calculate the unadjusted hazard ratio. Those with *P* ≤ 0.2 were chosen as potential confounding factors. These potential confounding factors were then added to the final multivariable regression model. Similarly, multivariable logistic regression analysis was used to analyze index admission mortality. Kaplan-Meier curves were constructed to visualize readmission rates over the 30-day follow-up period. All analyses accounted for the complex sampling design and weighting of the NRD to produce nationally representative estimates. Two-sided *P* < 0.05 was considered statistically significant. Analyses were performed using Stata (Version 17.0, College Station, Texas, United States).

## Results

### Baseline study population characteristics

Baseline demographics and hospital characteristics differed between the two groups ( Table 1 ). Patients undergoing ERCP-guided drainage were more likely to be female (51.60% vs 40.62%; *P* < 0.001), have private insurance (26.81% vs. 15.50%; *P* < 0.001), and have certain comorbidities such as hypertension (42.2% vs. 38.02%; *P* < 0.001), liver disease (8.04% vs. 5.64%; *P* < 0.001), and peptic ulcer disease (3.35% vs. 1.34%; *P* < 0.001). In contrast, the PTC group was older (mean age 70.97 vs. 63.01 years; *P* < 0.001) with higher rates of congestive heart failure (25.79% vs. 11.77%; *P* < 0.001) and peripheral vascular disease (5.02% vs. 2.42%; *P* < 0.001). The number of procedures increased over the study period, with ERCP-guided drainage rising from 798 cases in 2016 to 1,208 in 2019, and PTC increasing from 18,833 to 22,479 cases.

**Table:** **Table 1** Baseline demographics and hospital characteristics of acute cholecystitis patients undergoing ERCP-guided gallbladder drainage vs. percutaneous cholecystostomy.

	**ERCP-guided gallbladder drainage**	**Percutaneous cholecystostomy**
**Total**	N = 3592	N = 80,372
**Mean age**	63.01 (62.07 to 63.96)	70.97 (70.76 to 71.17)
**Gender**		
Male	48.40%	59.38%
Female	51.60%	40.62%
**Insurance**		
Medicare	56.27%	73.21%
Medicaid	12.57%	9.34%
Private insurance	26.81%	15.50%
Other insurance types	4.34%	1.94%
**Median household income**		
0 to 25th percentile	24.58%	25.94%
26th to 50th percentile	27.86%	26.32%
51st to 75th percentile	25.91%	25.97%
76th to 100th percentile	21.64%	21.77%
**Hospital bed size**		
Small	13.43%	14.97%
Medium	25.36%	26.88%
Large	61.22%	58.15%
**Hospital teaching status**		
Non-teaching	23.03%	20.12%
Teaching	76.97%	79.88%
**Hospital urban-rural location**		
Rural	1.73%	3.22%
Urban	98.27%	96.78%
**Comorbidities**		
Alcoholic liver disease	2.45%	2.52%
Congestive heart failure	11.77%	25.79%
Cardiac arrhythmia	7.66%	8.75%
Valvular disease	2.50%	2.84%
Peripheral vascular disease	2.42%	5.02%
Hypertension	42.20%	38.02%
Other neurological disorder	1.62%	3.16%
COPD	0.30%	0.40%
Type 2 diabetes mellitus	14.31%	14.64%
Type 2 diabetes mellitus with complication	0.21%	0.25%
Hypothyroidism	0.75%	0.76%
Liver disease including cirrhosis	8.04%	5.64%
Peptic ulcer disease excluding bleeding	3.35%	1.34%
HIV	0.24%	0.28%
History of solid tumor	1.25%	1.42%
Alcohol use disorder	0.41%	0.40%
**Patient location**		
Central counties metro areas of ≥ 1 million population	28.98%	28.28%
Fringe counties of metro areas of ≥ 1 million population	26.04%	27.37%
Counties in metro areas of 250,000–999,999 population	21.79%	21.33%
Counties in metro areas of 50,000–249,999 population	9.77%	8.82%
Micropolitan counties	7.41%	7.59%
Not metropolitan or micropolitan counties	6.00%	6.60%
**Admission day is a weekend**		
Admitted M-F	74.75%	74.40%
Admitted Saturday-Sunday	25.25%	25.60%
COPD, chronic obstructive pulmonary disease; ERCP, endoscopic retrograde cholangiopancreatography.		

### Hospital readmission

**Fig. 1:**
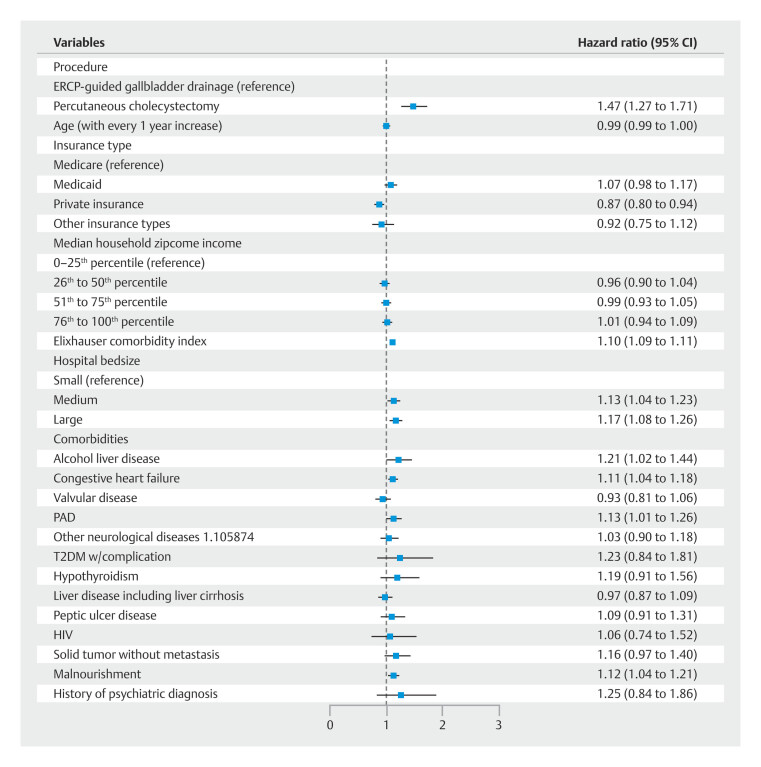
Adjusted hazard ratio for all-cause 30-day readmission among index admissions for Acute Cholecystitis.

**Fig. 2:**
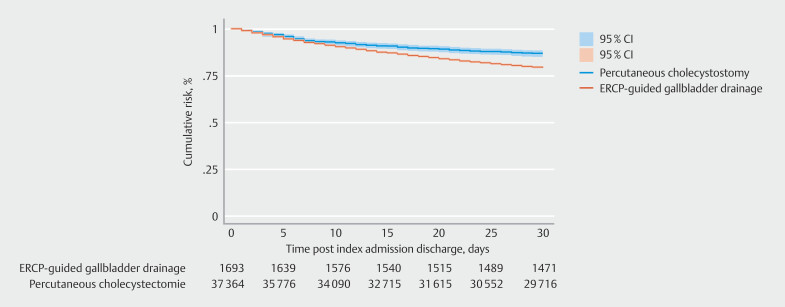
Kaplan-Meier curve of all-cause 30-day readmission comparing ERCP-guided gallbladder drainage and Percutaneous cholecystostomy.

In our study cohort, the PTC group had a significantly higher 30-day readmission rate compared with ERCP-guided drainage (20.67% vs. 12.76%; *P* < 0.001). Utilizing multivariable Cox regression analysis, compared with ERCP-guided gallbladder drainage, PTC had a higher 30-day readmission risk (adjusted hazard ratio 1.47; 95% confidence interval [CI] 1.27–1.71; *P* < 0.001) ( Fig. 1 and Fig. 2 ). Other notable clinical predictors for 30-day readmission included higher Elixhauser comorbidity index, alcoholic liver disease, and congestive heart failure.

Our propensity score-matched cohort compared patients who underwent PTC and ERCP-guided gallbladder drainage. The Charlson Comorbidity Index was also comparable, with no significant differences observed ( *P* = 0.932) ( **Supplementary Table 1** ). In this analysis, we observed that the all-cause readmission rate for the PTC group was 17.88% (95% CI 14.70–21.57), whereas the readmission rate for the ERCP-guided gallbladder drainage group was 12.47% (95% CI 9.78–15.78) ( **Supplementary Fig. 2** ).

### Morbidity and mortality

**Fig. 3:**
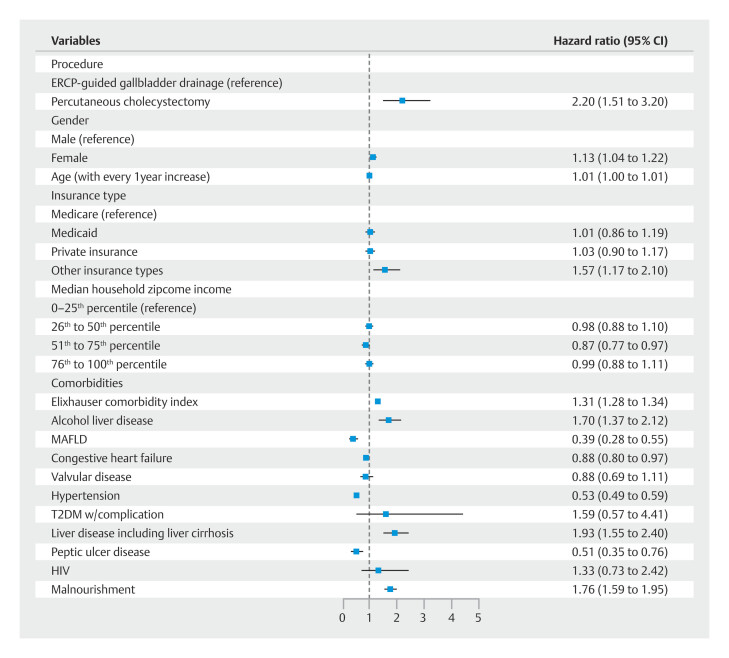
Multivariate logistic analysis for index admission mortality among patient with Acute cholecystitis that underwent percutaneous cholecystostomy or endoscopic-guided gallbladder drainage. T2DM, type 2 diabetes mellitus; MAFLD, metabolic fatty liver disease.

During index admission, PTC was associated with a higher mortality rate (8.15% vs 2.50%; *P* < 0.001). Using multivariate logistic regression analysis, we observed PTC had higher odds for index admission mortality (adjusted odds ratio: 2.20; 95% CI: 1.51 to 3.20; *P* < 0.001). Other predictors for index admission mortality are shown in Fig. 3 . ERCP-guided drainage was consistently associated with lower rates of in-hospital AEs compared with PTC. These included acute hypoxemic respiratory failure (6.69% vs 18.48%; *P* < 0.001), acute renal failure (18.22% vs 40.91%; *P* < 0.001), shock (0.47% vs 1.15%; *P* < 0.001), and need for blood transfusions (3.27% vs 8.13%; *P* < 0.001), vasopressors (1.25% vs 4.08%; *P* < 0.001), and mechanical ventilation (1.25% vs 4.08%; *P* < 0.001). The exception was a similar occurrence of lower gastrointestinal bleeding (11.98% vs 11.02%; *P* = 0.226) ( Table 2 ).

**Table:** **Table 2** Secondary outcomes with ERCP-guided gallbladder drainage and percutaneous cholecystectomy.

**Secondary outcome**	**ERCP-guided gallbladder drainage**	**Percutaneous cholecystostomy**	***P* value **
Index admission mortality	2.50%	8.15%	*P* < 0.001
Acute hypoxemic respiratory failure	6.69%	18.48%	*P* < 0.001
Acute renal failure	18.22%	40.91%	*P* < 0.001
Shock	0.47%	1.15%	*P* < 0.001
Requiring blood transfusion	3.27%	8.13%	*P* < 0.001
Lower gastrointestinal bleeding	11.98%	11.02%	*P* = 0.226
Vasopressor requirement	1.25%	4.08%	*P* < 0.001
Mechanical ventilation requirement	1.25%	4.08%	*P* < 0.001
30-day readmission rate	12.76%	20.67%	*P* < 0.001
30-day readmission for acute cholecystitis	0.86%	2.72%	*P* < 0.001
30-day readmission for acute cholangitis	1,16%	0.36%	*P* < 0.001
30-day readmission for biliary pancreatitis	0.24%	0.30%	*P* = 0.569
30-day readmission for choledocholithiasis	0.48%	0.60%	*P* = 0.473
30-day readmission for composite biliary events	2.78%	3.62%	*P* = 0.771
ERCP, endoscopic retrograde cholangiopancreatography.			

**Fig. 4:**
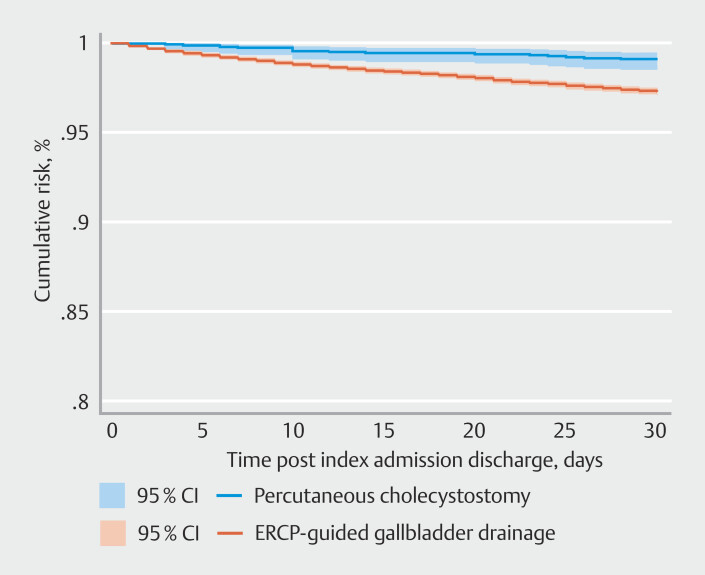
Kaplan-Meier curve of all-cause 30-day acute cholecystitis readmission comparing ERCP-guided gallbladder drainage and percutaneous cholecystostomy.

Readmission causes were analyzed for both ERCP-guided procedures and PTC. The overall rate of biliary-related readmissions was 2.78% (95% CI 2.10%-3.68%) for ERCP procedures and 3.62% (95% CI 3.42%-3.83%) for PTC. When examining specific conditions, cholangitis accounted for 1.16% of ERCP readmissions (95% CI 0.72–1.89%) and 0.36% of PTC readmissions (95% CI 0.30–0.43%). Acute biliary pancreatitis was responsible for 0.28% of ERCP readmissions (95% CI 0.13%-0.64%) and 0.24% of PTC readmissions (95% CI 0.19%-0.29%). Choledocholithiasis led to readmission in 0.56% of ERCP cases (95% CI 0.32%-1.00%) and 0.48% of PTC cases (95% CI 0.41%-0.57%). For acute cholecystitis, the readmission rate was 1.06% (95% CI 0.44%-2.52%) in the ERCP group compared with 3.53% (95% CI 2.20%-5.62%) in the PTC group, with Cox proportional hazards analysis showing a 3.41-fold increased risk (95% CI 1.99%-5.84) in the PTC group in reference to the ERCP-guided group ( Fig. 4 ).

In the propensity score-matched analysis, the 30-day readmission rate for acute cholecystitis was significantly different between the two procedures. For the ERCP-guided procedure, the readmission rate was 1.06% (95% CI 0.44–2.52). In contrast, the PTC group had a higher readmission rate of 3.53% (95% CI 2.20–5.62). These findings indicate a higher likelihood of 30-day readmissions for patients undergoing PTC compared with those receiving the ERCP-guided approach.

## Discussion

Our nationwide cohort study comparing ERCP-guided gallbladder drainage and PTC in patients with acute cholecystitis revealed several significant clinical findings. ERCP-guided drainage was associated with substantially lower 30-day readmission rates compared with PTC (12.76% vs. 20.67%, *P* < 0.001). After adjusting for potential confounders, PTC carried a 47% higher risk of 30-day readmission. In addition, ERCP-guided drainage demonstrated lower index admission mortality (2.50% vs. 8.15%, *P* < 0.001) and reduced rates of several inpatient AEs, including acute hypoxemic respiratory failure, acute renal failure, shock, and need for blood transfusions, vasopressors, and mechanical ventilation ( *P* < 0.001 for all comparisons). The overall rate of biliary-related readmissions was 2.78% (95% CI 2.10%-3.68%) for ERCP procedures and 3.62% (95% CI 3.42%-3.83%) for PTC. Notably, the PTC group had a significantly higher rate of readmission specifically for acute cholecystitis compared with the ERCP-guided drainage group (2.72% vs. 0.86%, *P* < 0.005), with a 3.41-fold increased risk as shown by Cox proportional hazards analysis.

These findings extend the existing literature on gallbladder drainage techniques for acute cholecystitis in high-risk patients. Our study addressed baseline differences between the two groups including multivariate analysis and propensity score matching. Notably, in the propensity-matched analysis, mean age of the two cohorts was not significantly different, suggesting that the observed outcome differences are less likely to be driven by age-related factors. This careful adjustment for baseline characteristics enhances the robustness of our results and supports the possibility that ERCP-guided drainage may offer superior clinical outcomes compared with PTC. Our results are consistent with some studies 5 8 , which reported significantly lower readmission rates with ERCP-guided drainage—a finding similar to our observations. However, they contrast with other studies 9 10 , which found no significant difference in readmission rates between endoscopic and percutaneous approaches. Despite these variations, there is a consistent trend across the literature showing lower reintervention rates associated with endoscopic approaches 7 11 12 .

The lower readmission rates for recurrent acute cholecystitis in the ERCP-guided group can be attributed to fundamental differences between the procedures. Percutaneous drainage typically involves an external catheter, prone to complications such as dislodgement, infection, and bile leakage 13 . In contrast, ERCP-guided transpapillary drainage creates an internal drainage pathway, eliminating issues associated with external tubes and allowing for more physiologic bile flow 14 . Siddiqui et al 8 . reported significantly lower unplanned hospital readmission rates with transpapillary drainage (3.2%) compared with percutaneous drainage (19.8%). This is supported by a recent meta-analysis 15 , which found a recurrent cholecystitis rate of only 3% for transpapillary drainage, compared with published rates of 22% for percutaneous drainage in other studies 16 .

In addition, transpapillary drainage requires fewer additional interventions. Siddiqui et al 8 found that only 11% of transpapillary drainage patients needed additional surgical intervention, versus 49% in the percutaneous group. The internal nature of transpapillary drainage also allows for longer-term management in poor surgical candidates. Studies have noted that plastic stents used in transpapillary drainage can remain patent for extended periods, with bile flowing both through and around the stent, potentially reducing recurrent cholecystitis risk 17 . These factors collectively contribute to better outcomes and lower unplanned readmission rates observed with ERCP-guided transpapillary drainage, making it a viable option for gallbladder drainage in high-risk patients with acute cholecystitis.

The clinical implications of our study are significant for management of acute cholecystitis in high-risk surgical candidates. To this end, patients with a higher comorbidity index were found to be at a higher risk for hospital readmission. Lisotti et al. reported a similar finding where patients with higher Charlson Comorbidity Index were found to be at a higher risk for long-term mortality after EUS-guided drainage 18 . Interestingly, the study found that this was independent from the clinical success rate for the procedure. Moreover, an Italian nationwide study of 116 patients found that despite a relatively high clinical success (87.1%) and low rate of AEs (10%), 30-day mortality was 19.8% (21/106) and the overall mortality rate during follow-up was 36.8% (39/106) 19 . The authors reported that most patients died due to underlying comorbidities including advanced malignancies, heart failure, renal and liver impairments. The aforementioned studies underscore the importance of accounting for patient comorbidities prior to procedure planning as well as the consent process.

Patient selection for ERCP-guided gallbladder drainage should consider comorbidities, risk of readmission, anesthesia tolerance, and potential for future cholecystectomy. Our findings of lower 30-day readmission rates and reduced AEs with ERCP-guided drainage suggest that patients at higher risk for readmission or recurrent cholecystitis, in particular, may benefit from this approach. Although those unable to tolerate general anesthesia might still require PTC under local anesthesia, ERCP-guided drainage offers potential advantages for subsequent surgical management.

Furthermore, literature indicates that endoscopic drainage techniques can lead to faster resolution of cholecystitis 20 . This may facilitate easier gallbladder dissection during future cholecystectomy, potentially reducing operative time and lowering risk of conversion to open surgery 20 21 . Implementation of ERCP-guided drainage requires a multidisciplinary approach and clinical providers should be aware of the learning curve associated with this technique 22 . Because our study demonstrates the benefits of ERCP-guided drainage in terms of reduced readmissions and complications, these additional surgical considerations further support its potential advantages in comprehensive patient care for acute cholecystitis.

Our study has several limitations inherent in its retrospective design and use of the NRD, which may impact interpretation of our main findings in several important ways. First, reliance on ICD-10 codes for identifying procedures and outcomes may lead to misclassification bias. Accuracy of coding can vary between institutions and may not capture the full clinical picture or procedure details, potentially leading to under-estimation or over-estimation of procedure rates and outcomes. This could affect the reported differences between ERCP-guided drainage and PTC. Second, the NRD lacks granular clinical data such as laboratory values, imaging findings, and severity scores for acute cholecystitis. This limitation means we cannot fully account for disease severity, which could confound the relationship between procedure choice and outcomes. Furthermore, such population-level studies do not account for multiple, repeat, or failed attempts at cannulating the cystic duct or concomitantly treating bile duct stones.

Patients with more severe cases might be more likely to undergo PTC, potentially biasing the results against this procedure. Third, we cannot account for operator experience or institutional expertise, which may significantly influence outcomes of endoscopic procedures. Finally, the reasons for choosing one procedure over another are not captured in the database, potentially introducing selection bias. However, despite these shortcomings, we used propensity scoring to a very large population-level database to provide estimates lacking in the current literature.

## Conclusions

In conclusion, this nationwide cohort study demonstrates that ERCP-guided gallbladder drainage is associated with significantly better outcomes compared with PTC in patients with acute cholecystitis. Key findings include lower 30-day readmission rates, lower index admission mortality, and fewer inpatient AEs with ERCP-guided drainage. The study also reveals a lower rate of readmission for recurrent acute cholecystitis in the ERCP-guided group. These results suggest that ERCP-guided drainage may be a superior alternative for managing acute cholecystitis in patients who are not surgical candidates.

## Supplementary Material

Supplementary Material Zusatzmaterial
