# Supplementary material for: Endoscopic transpapillary gallbladder stenting vs percutaneous cholecystostomy for managing acute cholecystitis: Nationwide propensity score study
Source: Endosc Int Open. 2025 Feb 26;13:a25210084. doi: 10.1055/a-2521-0084 (PMC11866036; doi:10.1055/a-2521-0084)

Supplementary Table 1 Comparison of baseline characteristics between propensity-matched patients undergoing ERCP-guided gallbladder drainage and percutaneous cholecystostomy for acute cholecystitis.

	**Percutaneous cholecystostomy**	**ERCP-guided gallbladder drainage**
Total number	N = 834	N = 829
Mean age	64.11 (95%CI 62.57-65.66)	63.04 (95%CI 61.38-64.69)
**Gender**		
Male	56.40%	48.50%
Female	43.60%	51.50%
**Insurance type**		
Medicaid	56.58%	55.45%
Medicare	15.68%	13.91%
Private	23.60%	25.38%
Other	4.14%	5.26%
**Median household income**		
0 to 25th percentile	21.23%	21.80%
26th to 50th percentile	25.95%	26.96%
51st to 75th percentile	29.04%	27.34%
76th to 100th percentile	23.77%	23.90%
**Hospital bed size**		
Small	9.73%	11.09%
Medium	26.85%	26.13%
Large	63.42%	62.78%
**Hospital teaching status**		
Non-teaching	23.42%	24.06%
Teaching	76.58%	75.94%
**Hospital urban-rural location**		
Rural	0.36%	1.13%
Urban	99.64%	98.87%
**Charlson Comorbidities Index**		
0	34.05%	35.71%
1	23.24%	23.31%
2	12.79%	12.59%
3	29.91%	28.38%

ERCP, endoscopic retrograde cholangiopancreatography.

Supplementary Fig. 1 Propensity score distributions before and after matching for endoscopic-guided and PTC groups.

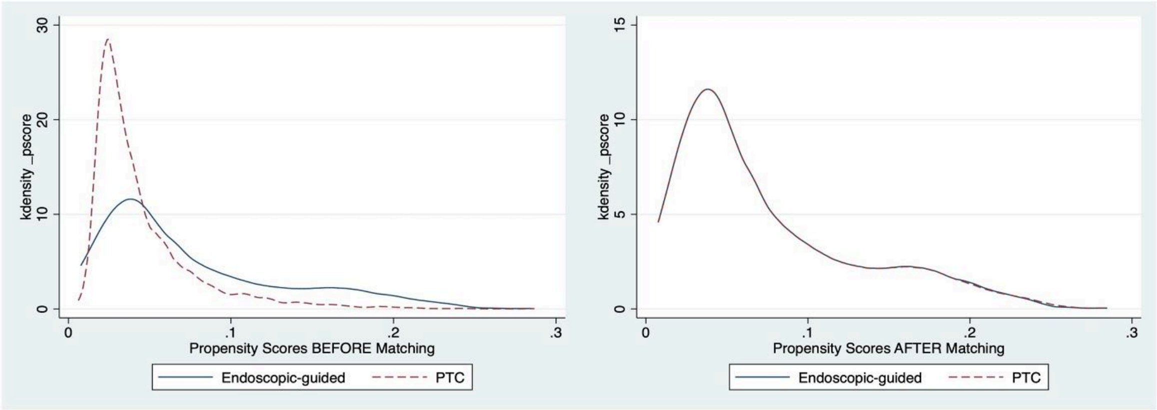

Supplementary Fig. 2 Kaplan-Meier curve of all-cause 30-day readmission comparing ERCP-guided gallbladder drainage and percutaneous cholecystostomy in the propensity-matched cohort.

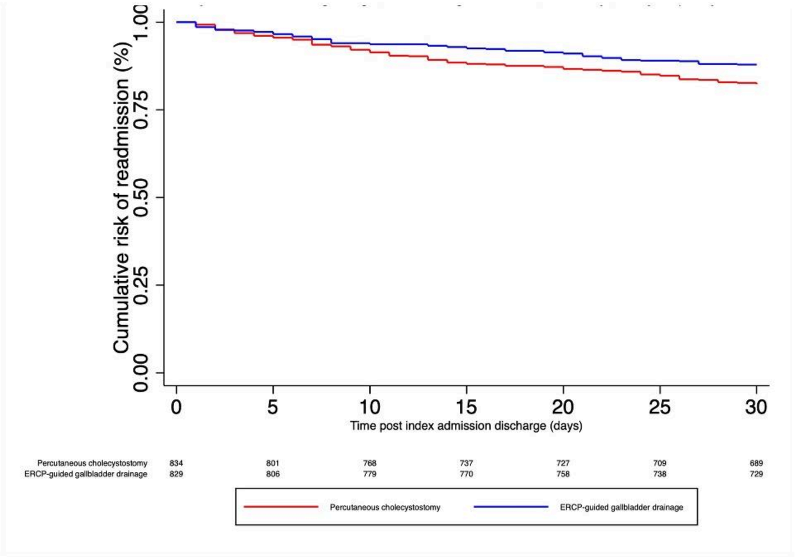

Supplement: Supplementary file 1 — Supplementary Material [file 10-1055-a-2521-0084_25221337.pdf]
